# {3,14-Dimethyl-2,6,13,17-tetra­aza­tricyclo­[16.4.0.0^7,12^]docosane-κ^4^ *N*,*N*′,*N*′′,*N*′′′)bis­(nitrato-κ*O*)copper(II)

**DOI:** 10.1107/S1600536812001845

**Published:** 2012-01-21

**Authors:** Jong-Ha Choi, Md Abdus Subhan, Seik Weng Ng

**Affiliations:** a Department of Chemistry, Andong National University, Andong 760-749, Republic of Korea; b Department of Chemistry, University of Malaya, 50603 Kuala Lumpur, Malaysia; c Chemistry Department, Faculty of Science, King Abdulaziz University, PO Box 80203 Jeddah, Saudi Arabia

## Abstract

The Cu^II^ atom in the title compound, [Cu(NO_3_)_2_(C_20_H_40_N_4_)], is *N*,*N*′,*N*′′,*N*′′′-chelated by the macrocyclic ligand: the four N atoms form a square, above and below which are located the O atoms of the nitrate ions. The metal atom exists in a tetra­gonally distorted octa­hedron, on a special position of 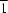 site symmetry. One of the amino groups is hydrogen bonded to an O atom of the nitrate ion. The other amino group is hydrogen bonded to O atom of an adjacent mol­ecule, generating a supra­molecular dimeric hydrogen-bonded dinuclear aggregate.

## Related literature

For the synthesis of the cyclam, see: Choi *et al.* (2012 ▶). For similar copper nitrate–cyclam adducts, see: Amadei *et al.* (1999 ▶); Choi *et al.* (2001 ▶, 2006 ▶); Dong *et al.* (1999 ▶); Liu & Chu (2010 ▶).
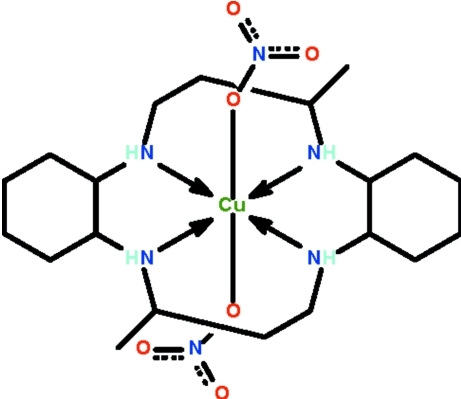


## Experimental

### 

#### Crystal data

[Cu(NO_3_)_2_(C_20_H_40_N_4_)]
*M* *_r_* = 524.12
Triclinic, 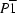

*a* = 8.2552 (10) Å
*b* = 8.8074 (11) Å
*c* = 9.1399 (10) Å
α = 67.879 (12)°
β = 68.780 (11)°
γ = 75.096 (11)°
*V* = 568.23 (12) Å^3^
*Z* = 1
Mo *K*α radiation
μ = 1.01 mm^−1^
*T* = 100 K
0.30 × 0.20 × 0.10 mm

#### Data collection

Agilent SuperNova Dual diffractometer with an Atlas detector
Absorption correction: multi-scan (*CrysAlis PRO*; Agilent, 2011) ▶ *T*_min_ = 0.751, *T*_max_ = 0.906
4122 measured reflections
2332 independent reflections
1963 reflections with *I* > 2σ(*I*)
*R*_int_ = 0.064

#### Refinement

*R*[*F* ^2^ > 2σ(*F* ^2^)] = 0.052
*wR*(*F* ^2^) = 0.132
*S* = 1.02
2332 reflections
159 parameters
2 restraints
H atoms treated by a mixture of independent and constrained refinement
Δρ_max_ = 0.96 e Å^−3^
Δρ_min_ = −0.68 e Å^−3^

### 

Data collection: *CrysAlis PRO* (Agilent, 2011 ▶); cell refinement: *CrysAlis PRO*; data reduction: *CrysAlis PRO*; program(s) used to solve structure: *SHELXS97* (Sheldrick, 2008 ▶); program(s) used to refine structure: *SHELXL97* (Sheldrick, 2008 ▶); molecular graphics: *X-SEED* (Barbour, 2001 ▶); software used to prepare material for publication: *publCIF* (Westrip, 2010 ▶).

## Supplementary Material

Crystal structure: contains datablock(s) global, I. DOI: 10.1107/S1600536812001845/xu5440sup1.cif

Structure factors: contains datablock(s) I. DOI: 10.1107/S1600536812001845/xu5440Isup2.hkl

Additional supplementary materials:  crystallographic information; 3D view; checkCIF report

## Figures and Tables

**Table 1:** Hydrogen-bond geometry (Å, °)

*D*—H⋯*A*	*D*—H	H⋯*A*	*D*⋯*A*	*D*—H⋯*A*
N1—H1⋯O2^i^	0.88 (1)	2.15 (2)	2.992 (3)	160 (3)
N2—H2⋯O3^ii^	0.88 (1)	2.23 (2)	2.961 (3)	140 (3)

Symmetry codes: (i) 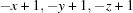; (ii) 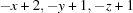.

**Fig. 1.**
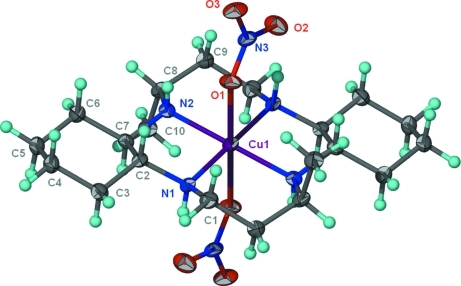
Thermal ellipsoid plot (Barbour, 2001) of Cu(NO3)2(C20H40N4) at the 70% probability level; hydrogen atoms are drawn as spheres of arbitrary radius.

## supplementary crystallographic information

### Comment

The macrocycle, cyclam (1,4,8,11-tetraazacyclotetradecane), forms a large number of complexes with copper(II) salts in which the macrocle chelates in a tetradentate matter. In same cases, the counterion bonded to the metal atoms and in other cases, the metal atom exists in square-pyramidal geometry as the counterion is far away. The crystal structure of copper nitrate–cyclam has not been reported; the crystal structures of other substituted cyclams have the metal atom in a tetragonally elongated octahedral geometry (Amadei *et al.*, 1999; Choi *et al.*, 2006; Choi *et al.*, 2001; Dong *et al.*, 1999; Liu & Chu, 2010). The Cu^II^ atom in the title compound (Scheme I) is similarly chelated by the macrocyclic ligand in a tetragonally distorted octahedron (Fig.1). The atom lies on a special position of –*1* site symmetry. One of the amino groups is hydrogen-bonded to an O atom of the nitrate ion. The other amino group is hydrogen-bonded to O atom of an adjacent molecule to generate a hydrogen-bonded dinuclear molecule (Table 1).

### Experimental

The macrocycle co-crystal, 3,14-dimethyl-2,6,13,17-tetraazatricyclo(16.4.0.0^7,12^)docosane (naphthalen-1-yl)methanol prepared as described (Choi *et al.*, 2012). Copper nitrate trihydrate (0.242 g, 1 mmol) dissolved in methanol (10 ml) was mixed with a suspension of the macrocycle co-crystal (0.163 g, 2.5 mmol) dissolved in methanol (10 ml). The mixture was heated for 30 minutes and then set aside for the growth of purple crystals.

### Refinement

Carbon-bound H-atoms were placed in calculated positions [C–H 0.99 to 1.00 Å, *U*_iso_(H) 1.2 to 1.5*U*_eq_(C)] and were included in the refinement in the riding model approximation.

The amino H-atoms were located in a difference Fourier map, and were refined with a distance restraint of N–H 0.88±0.01 Å; their temperature factors were refined.

### Crystal data

**Table:**

[Cu(NO_3_)_2_(C_20_H_40_N_4_)]	*Z* = 1
*M**_r_* = 524.12	*F*(000) = 279
Triclinic, *P*1	*D*_x_ = 1.532 Mg m^−^^3^
Hall symbol: -P 1	Mo *K*α radiation, λ = 0.71073 Å
*a* = 8.2552 (10) Å	Cell parameters from 1668 reflections
*b* = 8.8074 (11) Å	θ = 2.5–27.5°
*c* = 9.1399 (10) Å	µ = 1.01 mm^−^^1^
α = 67.879 (12)°	*T* = 100 K
β = 68.780 (11)°	Prism, purple
γ = 75.096 (11)°	0.30 × 0.20 × 0.10 mm
*V* = 568.23 (12) Å^3^	

### Data collection

**Table:**

Agilent SuperNova Dual diffractometer with an Atlas detector	2332 independent reflections
Radiation source: SuperNova (Mo) X-ray Source	1963 reflections with *I* > 2σ(*I*)
Mirror	*R*_int_ = 0.064
Detector resolution: 10.4041 pixels mm^-1^	θ_max_ = 26.5°, θ_min_ = 2.5°
ω scan	*h* = −7→10
Absorption correction: multi-scan (CrysAlis PRO; Agilent, 2011)	*k* = −10→11
*T*_min_ = 0.751, *T*_max_ = 0.906	*l* = −11→10
4122 measured reflections	

### Refinement

**Table:**

Refinement on *F*^2^	Primary atom site location: structure-invariant direct methods
Least-squares matrix: full	Secondary atom site location: difference Fourier map
*R*[*F*^2^ > 2σ(*F*^2^)] = 0.052	Hydrogen site location: inferred from neighbouring sites
*wR*(*F*^2^) = 0.132	H atoms treated by a mixture of independent and constrained refinement
*S* = 1.02	*w* = 1/[σ^2^(*F*_o_^2^) + (0.0874*P*)^2^] where *P* = (*F*_o_^2^ + 2*F*_c_^2^)/3
2332 reflections	(Δ/σ)_max_ = 0.001
159 parameters	Δρ_max_ = 0.96 e Å^−^^3^
2 restraints	Δρ_min_ = −0.68 e Å^−^^3^

### Fractional atomic coordinates and isotropic or equivalent isotropic displacement parameters (Å^2^)

**Table:**

	*x*	*y*	*z*	*U*_iso_*/*U*_eq_	
Cu1	0.5000	0.5000	0.5000	0.0158 (2)	
N1	0.5570 (3)	0.5225 (3)	0.2605 (3)	0.0152 (5)	
H1	0.473 (3)	0.481 (4)	0.257 (4)	0.022 (9)*	
N2	0.6852 (3)	0.2945 (3)	0.5073 (3)	0.0154 (5)	
H2	0.777 (3)	0.342 (3)	0.484 (4)	0.014 (8)*	
N3	0.8305 (3)	0.6621 (3)	0.5099 (3)	0.0193 (6)	
O1	0.7574 (3)	0.6375 (3)	0.4223 (3)	0.0228 (5)	
O2	0.7518 (3)	0.6441 (3)	0.6596 (3)	0.0280 (5)	
O3	0.9780 (3)	0.7070 (3)	0.4452 (3)	0.0260 (5)	
C1	0.5512 (4)	0.6927 (4)	0.1448 (4)	0.0201 (6)	
H1A	0.5787	0.6891	0.0312	0.024*	
H1B	0.6413	0.7475	0.1460	0.024*	
C2	0.7261 (4)	0.4161 (4)	0.2131 (4)	0.0171 (6)	
H2A	0.8227	0.4708	0.2045	0.020*	
C3	0.7647 (4)	0.3870 (4)	0.0493 (4)	0.0203 (6)	
H3A	0.7728	0.4940	−0.0408	0.024*	
H3B	0.6675	0.3379	0.0528	0.024*	
C4	0.9369 (4)	0.2708 (4)	0.0141 (4)	0.0207 (6)	
H4A	0.9581	0.2509	−0.0913	0.025*	
H4B	1.0353	0.3236	0.0023	0.025*	
C5	0.9308 (4)	0.1064 (4)	0.1531 (4)	0.0206 (6)	
H5A	1.0448	0.0348	0.1299	0.025*	
H5B	0.8386	0.0494	0.1593	0.025*	
C6	0.8921 (4)	0.1347 (4)	0.3181 (4)	0.0196 (6)	
H6A	0.9906	0.1813	0.3155	0.024*	
H6B	0.8821	0.0275	0.4079	0.024*	
C7	0.7216 (4)	0.2533 (4)	0.3538 (4)	0.0169 (6)	
H7	0.6221	0.2001	0.3659	0.020*	
C8	0.6715 (4)	0.1477 (4)	0.6603 (4)	0.0177 (6)	
H8	0.7896	0.0787	0.6487	0.021*	
C9	0.6290 (4)	0.2073 (4)	0.8083 (4)	0.0192 (6)	
H9A	0.6398	0.1093	0.9053	0.023*	
H9B	0.7189	0.2758	0.7851	0.023*	
C10	0.5417 (4)	0.0404 (4)	0.6826 (4)	0.0206 (6)	
H10A	0.5752	0.0052	0.5846	0.031*	
H10B	0.4238	0.1039	0.6971	0.031*	
H10C	0.5422	−0.0573	0.7804	0.031*	

### Atomic displacement parameters (Å^2^)

**Table:**

	*U*^11^	*U*^22^	*U*^33^	*U*^12^	*U*^13^	*U*^23^
Cu1	0.0102 (3)	0.0195 (3)	0.0187 (3)	−0.00213 (18)	−0.0034 (2)	−0.0080 (2)
N1	0.0079 (11)	0.0190 (13)	0.0199 (13)	−0.0024 (9)	−0.0025 (10)	−0.0087 (10)
N2	0.0119 (12)	0.0181 (13)	0.0187 (12)	−0.0057 (9)	−0.0030 (10)	−0.0077 (10)
N3	0.0125 (12)	0.0193 (13)	0.0287 (15)	−0.0028 (9)	−0.0085 (11)	−0.0076 (11)
O1	0.0175 (11)	0.0303 (12)	0.0264 (11)	−0.0086 (9)	−0.0087 (9)	−0.0097 (10)
O2	0.0239 (12)	0.0397 (14)	0.0253 (12)	−0.0131 (10)	−0.0032 (10)	−0.0138 (10)
O3	0.0137 (11)	0.0310 (13)	0.0362 (13)	−0.0103 (9)	−0.0086 (10)	−0.0078 (10)
C1	0.0147 (14)	0.0264 (17)	0.0190 (15)	−0.0034 (12)	−0.0033 (12)	−0.0084 (13)
C2	0.0079 (13)	0.0242 (16)	0.0223 (15)	−0.0026 (11)	−0.0026 (11)	−0.0122 (13)
C3	0.0184 (15)	0.0252 (17)	0.0207 (15)	−0.0028 (12)	−0.0053 (12)	−0.0115 (13)
C4	0.0163 (15)	0.0252 (16)	0.0216 (15)	−0.0010 (12)	−0.0021 (12)	−0.0131 (13)
C5	0.0155 (15)	0.0241 (16)	0.0258 (16)	−0.0010 (12)	−0.0039 (12)	−0.0151 (13)
C6	0.0146 (14)	0.0233 (16)	0.0224 (15)	−0.0003 (11)	−0.0047 (12)	−0.0110 (13)
C7	0.0123 (14)	0.0232 (15)	0.0197 (15)	−0.0042 (11)	−0.0033 (11)	−0.0120 (12)
C8	0.0117 (14)	0.0210 (15)	0.0196 (15)	−0.0015 (11)	−0.0049 (11)	−0.0057 (12)
C9	0.0174 (15)	0.0215 (15)	0.0198 (15)	−0.0012 (11)	−0.0067 (12)	−0.0077 (12)
C10	0.0163 (15)	0.0216 (16)	0.0253 (16)	−0.0057 (11)	−0.0048 (12)	−0.0081 (13)

### Geometric parameters (Å, °)

**Table:**

Cu1—N1	2.007 (2)	C3—H3A	0.9900
Cu1—N1^i^	2.007 (2)	C3—H3B	0.9900
Cu1—N2^i^	2.044 (2)	C4—C5	1.523 (4)
Cu1—N2	2.044 (2)	C4—H4A	0.9900
Cu1—O1	2.463 (2)	C4—H4B	0.9900
N1—C1	1.475 (4)	C5—C6	1.527 (4)
N1—C2	1.486 (3)	C5—H5A	0.9900
N1—H1	0.879 (10)	C5—H5B	0.9900
N2—C7	1.487 (4)	C6—C7	1.532 (4)
N2—C8	1.496 (4)	C6—H6A	0.9900
N2—H2	0.878 (10)	C6—H6B	0.9900
N3—O3	1.241 (3)	C7—H7	1.0000
N3—O2	1.251 (3)	C8—C10	1.517 (4)
N3—O1	1.265 (3)	C8—C9	1.525 (4)
C1—C9^i^	1.524 (4)	C8—H8	1.0000
C1—H1A	0.9900	C9—C1^i^	1.524 (4)
C1—H1B	0.9900	C9—H9A	0.9900
C2—C3	1.520 (4)	C9—H9B	0.9900
C2—C7	1.521 (4)	C10—H10A	0.9800
C2—H2A	1.0000	C10—H10B	0.9800
C3—C4	1.530 (4)	C10—H10C	0.9800
			
N1—Cu1—N1^i^	180.0	H3A—C3—H3B	108.1
N1—Cu1—N2^i^	95.20 (9)	C5—C4—C3	110.9 (2)
N1^i^—Cu1—N2^i^	84.80 (9)	C5—C4—H4A	109.5
N1—Cu1—N2	84.80 (9)	C3—C4—H4A	109.5
N1^i^—Cu1—N2	95.20 (9)	C5—C4—H4B	109.5
N2^i^—Cu1—N2	180.000 (1)	C3—C4—H4B	109.5
N1—Cu1—O1	87.75 (8)	H4A—C4—H4B	108.1
N1^i^—Cu1—O1	92.25 (8)	C4—C5—C6	110.4 (2)
N2^i^—Cu1—O1	97.63 (8)	C4—C5—H5A	109.6
N2—Cu1—O1	82.37 (8)	C6—C5—H5A	109.6
C1—N1—C2	113.1 (2)	C4—C5—H5B	109.6
C1—N1—Cu1	116.33 (18)	C6—C5—H5B	109.6
C2—N1—Cu1	108.35 (17)	H5A—C5—H5B	108.1
C1—N1—H1	106 (2)	C5—C6—C7	111.2 (2)
C2—N1—H1	108 (2)	C5—C6—H6A	109.4
Cu1—N1—H1	104 (2)	C7—C6—H6A	109.4
C7—N2—C8	114.3 (2)	C5—C6—H6B	109.4
C7—N2—Cu1	107.58 (17)	C7—C6—H6B	109.4
C8—N2—Cu1	121.32 (18)	H6A—C6—H6B	108.0
C7—N2—H2	102 (2)	N2—C7—C2	107.0 (2)
C8—N2—H2	111 (2)	N2—C7—C6	113.1 (2)
Cu1—N2—H2	98 (2)	C2—C7—C6	111.3 (2)
O3—N3—O2	120.9 (2)	N2—C7—H7	108.4
O3—N3—O1	119.5 (2)	C2—C7—H7	108.4
O2—N3—O1	119.6 (2)	C6—C7—H7	108.4
N3—O1—Cu1	131.16 (18)	N2—C8—C10	112.4 (2)
N1—C1—C9^i^	111.0 (2)	N2—C8—C9	108.9 (2)
N1—C1—H1A	109.4	C10—C8—C9	112.8 (3)
C9^i^—C1—H1A	109.4	N2—C8—H8	107.5
N1—C1—H1B	109.4	C10—C8—H8	107.5
C9^i^—C1—H1B	109.4	C9—C8—H8	107.5
H1A—C1—H1B	108.0	C1^i^—C9—C8	116.4 (2)
N1—C2—C3	114.3 (2)	C1^i^—C9—H9A	108.2
N1—C2—C7	107.1 (2)	C8—C9—H9A	108.2
C3—C2—C7	111.1 (2)	C1^i^—C9—H9B	108.2
N1—C2—H2A	108.0	C8—C9—H9B	108.2
C3—C2—H2A	108.0	H9A—C9—H9B	107.3
C7—C2—H2A	108.0	C8—C10—H10A	109.5
C2—C3—C4	110.7 (2)	C8—C10—H10B	109.5
C2—C3—H3A	109.5	H10A—C10—H10B	109.5
C4—C3—H3A	109.5	C8—C10—H10C	109.5
C2—C3—H3B	109.5	H10A—C10—H10C	109.5
C4—C3—H3B	109.5	H10B—C10—H10C	109.5
			
N2^i^—Cu1—N1—C1	35.3 (2)	Cu1—N1—C2—C7	42.3 (2)
N2—Cu1—N1—C1	−144.7 (2)	N1—C2—C3—C4	−177.7 (2)
O1—Cu1—N1—C1	−62.11 (19)	C7—C2—C3—C4	−56.3 (3)
N2^i^—Cu1—N1—C2	164.12 (17)	C2—C3—C4—C5	57.5 (3)
N2—Cu1—N1—C2	−15.88 (17)	C3—C4—C5—C6	−57.2 (3)
O1—Cu1—N1—C2	66.66 (18)	C4—C5—C6—C7	56.0 (3)
N1—Cu1—N2—C7	−14.16 (18)	C8—N2—C7—C2	178.5 (2)
N1^i^—Cu1—N2—C7	165.84 (18)	Cu1—N2—C7—C2	40.6 (2)
O1—Cu1—N2—C7	−102.58 (18)	C8—N2—C7—C6	−58.6 (3)
N1—Cu1—N2—C8	−148.4 (2)	Cu1—N2—C7—C6	163.59 (19)
N1^i^—Cu1—N2—C8	31.6 (2)	N1—C2—C7—N2	−55.2 (3)
O1—Cu1—N2—C8	123.1 (2)	C3—C2—C7—N2	179.4 (2)
O3—N3—O1—Cu1	166.62 (19)	N1—C2—C7—C6	−179.2 (2)
O2—N3—O1—Cu1	−14.8 (4)	C3—C2—C7—C6	55.3 (3)
N1—Cu1—O1—N3	−172.7 (2)	C5—C6—C7—N2	−175.7 (2)
N1^i^—Cu1—O1—N3	7.3 (2)	C5—C6—C7—C2	−55.2 (3)
N2^i^—Cu1—O1—N3	92.4 (2)	C7—N2—C8—C10	−52.7 (3)
N2—Cu1—O1—N3	−87.6 (2)	Cu1—N2—C8—C10	78.8 (3)
C2—N1—C1—C9^i^	175.8 (2)	C7—N2—C8—C9	−178.4 (2)
Cu1—N1—C1—C9^i^	−57.8 (3)	Cu1—N2—C8—C9	−46.9 (3)
C1—N1—C2—C3	−63.6 (3)	N2—C8—C9—C1^i^	67.3 (3)
Cu1—N1—C2—C3	165.83 (19)	C10—C8—C9—C1^i^	−58.2 (3)
C1—N1—C2—C7	172.8 (2)		

Symmetry codes: (i) −*x*+1, −*y*+1, −*z*+1.

### Hydrogen-bond geometry (Å, °)

**Table:**

*D*—H···*A*	*D*—H	H···*A*	*D*···*A*	*D*—H···*A*
N1—H1···O2^i^	0.88 (1)	2.15 (2)	2.992 (3)	160 (3)
N2—H2···O3^ii^	0.88 (1)	2.23 (2)	2.961 (3)	140 (3)

Symmetry codes: (i) −*x*+1, −*y*+1, −*z*+1; (ii) −*x*+2, −*y*+1, −*z*+1.
